# Estimates of SARS-CoV-2 Hospitalization and Fatality Rates in the Prevaccination Period, United States

**DOI:** 10.3201/eid3006.231285

**Published:** 2024-06

**Authors:** Isabel Griffin, Jessica King, B. Casey Lyons, Alyson L. Singleton, Xidong Deng, Beau B. Bruce, Patricia M. Griffin

**Affiliations:** Centers for Disease Control and Prevention, Atlanta, Georgia, USA (I. Griffin, J. King, B.C. Lyons, X. Deng, B.B. Bruce, P.M. Griffin); Oak Ridge Institute for Science and Education, Oak Ridge, Tennessee, USA (A.L. Singleton)

**Keywords:** COVID-19, 2019 novel coronavirus disease, severe acute respiratory syndrome coronavirus 2, SARS-CoV-2, hospitalization, viruses, vaccine-preventable diseases, United States

## Abstract

Few precise estimates of hospitalization and fatality rates from COVID-19 exist for naive populations, especially within demographic subgroups. We estimated rates among persons with SARS-CoV-2 infection in the United States during May 1–December 1, 2020, before vaccines became available. Both rates generally increased with age; fatality rates were highest for persons >85 years of age (24%) and lowest for children 1–14 years of age (0.01%). Age-adjusted case hospitalization rates were highest for African American or Black, not Hispanic persons (14%), and case-fatality rates were highest for Asian or Pacific Islander, not Hispanic persons (4.4%). Eighteen percent of hospitalized patients and 44.2% of those admitted to an intensive care unit died. Male patients had higher hospitalization (6.2% vs. 5.2%) and fatality rates (1.9% vs. 1.5%) than female patients. These findings highlight the importance of collecting surveillance data to devise appropriate control measures for persons in underserved racial/ethnic groups and older adults.

The COVID-19 pandemic caused by the novel coronavirus SARS-CoV-2 resulted in >20 million reported cases, 480,000 hospitalizations, and 350,000 deaths in the United States through December 2020 (*1*). SARS-CoV-2 self-testing was not widely available at the time, which likely resulted in underreporting of infections (*2*,*3*). Beginning in mid-December 2020, COVID-19 case detection was affected by the availability of commercial testing and the introduction of at-home antigen-based diagnostic tests (*2*). The development of effective vaccines against SARS-CoV-2 after mid-December 2020 had notable effects on reducing hospitalization and fatality rates (*4*–*6*). Analyses in the United States and other countries have found higher case fatality rates associated with certain person-level (e.g., vaccination status, older age, race and ethnicity, and presence of underlying conditions), clinical (hospitalization and admission to critical care units), and country-level infrastructure (e.g., healthcare capacity) characteristics (*7*–*12*). Few precise estimates of hospitalization and mortality rates exist in the COVID-19–naive population in the United States, especially among demographic and clinical subgroups. We estimated US case hospitalization and fatality rates by demographic and clinical characteristics during May 1–December 1, 2020, before vaccine availability, among persons with reported SARS-CoV-2 infections. These estimates are unique because most populations worldwide now have some vaccine- or natural-induced immunity (*13*).

## Methods

State and territorial epidemiologists from 56 US jurisdictions submitted COVID-19 case reports to the Centers for Disease Control and Prevention (CDC) using a standard COVID-19 case report form; submissions were through direct data entry or comma separated value (CSV) files upload into CDC’s Data Collation and Integration for Public Health Event Response HHS Protect platform or through the National Notifiable Diseases Surveillance System (*14*–*16*). Jurisdictions included all 50 states, New York City (reporting separately from New York state), the District of Columbia (DC), and 4 territories (Guam, Northern Mariana Islands, Puerto Rico, and the US Virgin Islands). Jurisdictions voluntarily reported confirmed and probable COVID-19 cases based on the case definition promulgated by the Council of State and Territorial Epidemiologists (CSTE) at the time of reporting (*15*,*16*) (Appendix). Health officials in each jurisdiction determine the manner in which they obtain race and ethnicity information; common methods include interviews with patients and their families and reviews of medical records.

To create the hospitalization dataset, we identified jurisdictions in which >80% of case reports had the CDC hospitalization query not missing (i.e., answered yes, no, or unknown) for illnesses during May 1–December 1, 2020. If the date of illness onset was not provided or the person had no symptoms, the earliest date provided was used; this date was typically either the date the positive specimen was collected or the date of the case report. We used the same restriction to create the deaths dataset, requiring >80% of case reports with the CDC death query not missing for illnesses during May 1–December 1, 2020. This approach resulted in some variation in jurisdictions included in the hospitalization and death datasets. We then excluded from the hospitalization database any case record with missing information on hospitalization and excluded from the death database any case record with missing information on death.

For the hospitalization rate calculations, all numerators included confirmed and probable COVID-19 cases according to the CSTE definition at the time of reporting (Appendix). We calculated a lower bound for the rate by including in the denominator as not hospitalized those cases with unknown checked for hospitalization on the reporting form (N1 = 2,479,423). We calculated an upper bound for the rate by excluding from the denominator cases with unknown checked for hospitalization (n1 = 687,527 [27.7% of N1]). For the death rate calculations, all numerators included confirmed and probable COVID-19 cases according to the CSTE definition at the time of reporting (Appendix). We calculated a lower bound for the rate by including in the denominator as alive those cases with unknown checked for death on the reporting form (N2 = 4,708,444). We calculated an upper bound for the rate by excluding from the denominator cases with unknown checked for death (n2 = 756,133 [16.1% of N2]).

We chose a study period that reduced possible biases from variability in testing availability and data completeness. For each jurisdiction in the hospitalization analysis, we examined 2 metrics by week: the proportion of CDC’s COVID Electronic Lab Reporting (CELR) weekly testing volume reported as positive, and the proportion of cases reported to CDC that had symptoms using COVID-19 case report data. Those metrics had stabilized by May 1 and remained stable until December 1, 2020, which we chose as the last date included to eliminate any effects from vaccination. A brief survey was sent to health officials in jurisdictions included in the analysis to assess how data were being collected and reported.

We calculated case-hospitalization and case-fatality rates by sex (male, female), age group (<1, 1–4, 5–14, 15–24, 25–34, 35–44, 45–54, 55–64, 65–74, 75–84, and >85 years), race and ethnicity (American Indian or Alaska Native, not Hispanic or Latino; Asian or Pacific Islander, not Hispanic or Latino; African American or Black, not Hispanic or Latino; Hispanic; other or multiple races, not Hispanic or Latino; White, not Hispanic or Latino; and unknown), hospitalization and intensive care unit (ICU) admission (admitted to ICU; hospitalized but not admitted to ICU; and not hospitalized), and presence of any symptoms. We show race and ethnicity data in a combined variable; the other or multiple races category includes not Hispanic or Latino persons whose race was reported as other or for whom more than one race was reported, and persons whose record had both a racial designation and racial information unknown selected. The unknown category includes cases who have a known ethnicity of not Hispanic or Latino but either unknown or missing race; have a known race but either unknown or missing ethnicity; or have both unknown or missing race and unknown or missing ethnicity. Although included in the unknown category in the main analysis, we excluded persons from the race and ethnicity subanalysis if ethnicity was unknown, regardless of race.

To account for differences in age distribution by race and ethnicity, so rates among the groups can be compared, we adjusted hospitalization and fatality rates for race and ethnicity to the age distribution of the largest group in the 2019 national census of the US population: White, not Hispanic or Latino (*17*). We compared the census population distributions by sex, age group, and race and ethnicity for our subset populations to the full 2019 US census to determine whether our subsets were representative of the United States (Appendix Table 1) (*17*). We also describe demographic characteristics of all COVID-19 cases reported to CDC during May 1–December 1, 2020, by sex, age group, race and ethnicity, hospitalization and ICU admission status, presence of any symptoms, and deaths (Appendix Table 2). We used the 2019 US Census midyear estimates because they were the most recent data available at time of analysis.

### Ethics

This activity was deemed not to be research as defined in 45 CFR 46.102(l), and institutional review board review was not required. This activity was reviewed by CDC and was conducted consistent with applicable federal law and CDC policy (Appendix).

## Results

### Study Population and Comparison with Cases in the Entire United States

A total of 10,332,323 COVID-19 cases were reported to CDC during May 1, 2020–December 1, 2020. Only 58.8.% of those had valid, nonmissing information for hospitalization and 63.5% for death during the study period (Appendix Table 2), compared with >80% of records in the populations from which our datasets were drawn. After deleting records with hospitalization information missing, 2,479,423 cases from 21 jurisdictions were included in the hospitalization dataset (Table 1). After deleting records with death information missing, 4,708,444 cases from 22 jurisdictions were included in the deaths dataset (Table 2). The underlying populations of the study jurisdictions closely matched the US Census total population distribution in 2019 by sex and age group but varied by race and ethnicity (Appendix Table 1). The case-hospitalization dataset covers 25.5% of the US population, and the case-fatality dataset covers 43.7% of the US population.

**Table 1:** Demographic and clinical characteristics of SARS-CoV-2 infections that met inclusion criteria for case hospitalization rate analysis, United States, 2020*

Characteristic	Includes cases for which hospitalization status was unknown, counted as not hospitalized				Includes only cases for which hospitalization status was known		
	No. hospitalizations	No. cases	Case-hospitalization rate, %		No. hospitalizations	No. cases	Case-hospitalization rate, %
Overall	140,644	2,479,423	5.7		140,644	1,791,896	7.9
Sex							
M	72,372	1,164,257	6.2		72,372	834,733	8.7
F	67,894	1,300,788	5.2		67,894	946,921	7.2
Other	2	18	11.1		2	12	16.7
Missing†	134	4,006	3.3		134	2,657	5.0
Unknown	242	10,354	2.3		242	7,573	3.2
Age group, y							
<1	564	11,208	5.0		564	8,478	6.7
1–4	386	32,180	1.2		386	24,791	1.6
5–14	823	137,646	0.6		823	104,483	0.8
15–24	4,228	470,395	0.9		4,228	337,187	1.3
25–34	8,035	429,855	1.9		8,035	305,416	2.6
35–44	10,391	373,602	2.8		10,391	270,249	3.8
45–54	16,723	357,683	4.7		16,723	257,484	6.5
55–64	25,361	309,488	8.2		25,361	221,684	11.4
65–74	31,181	190,946	16.3		31,181	141,469	22.0
75–84	26,967	103,990	25.9		26,967	77,281	34.9
>85	15,973	61,604	25.9		15,973	42,872	37.3
Missing†	12	826	1.5		12	502	2.4
Race and ethnicity†§							
AI or AN, NH	1,974	26,745	7.4		1,974	21,821	9.1
Asian or PI, NH	3,468	49,774	7.0		3,468	43,118	8.0
AA or Black, NH	18,470	161,642	11.4		18,470	133,485	13.8
Hispanic	17,159	365,476	4.7		17,159	304,239	5.6
Other or multiple races, NH	4,705	60,408	7.8		4,705	47,583	9.9
White, NH	69,165	1,068,295	6.5		69,165	870,193	8.0
Unknown	25,703	747,083	3.4		25,703	371,457	6.9
Symptom status							
Symptomatic	105,131	1,664,240	6.3		105,131	1,484,676	7.1
Asymptomatic	1,870	55,987	3.3		1,870	50,657	3.7
Missing†	11,423	168,365	6.8		11,423	82,936	13.8
Unknown	22,220	590,831	3.8		22,220	173,627	12.8

*Data from 21 jurisdictions that met the study inclusion criteria, Case Only Epi Task Force dataset, accessed March 17, 2021, based on responses to the CDC 2019 Novel Coronavirus Case Report Form during May 1–December 1, 2020 (Figure). Reports in which no response was provided about hospitalization were excluded from the rate calculation. AA, African American; AI, American Indian; AN, Alaska Native; NH, not Hispanic or Latino; PI, Pacific Islander. †Missing indicates that the field was left blank. ‡Other or multiple races category includes NH persons whose race was reported as other or for whom >1 race was reported and persons whose record had both a racial designation and “racial information unknown” selected. The unknown category includes cases who have a known ethnicity of NH but either unknown or missing race; have a known race but either unknown or missing ethnicity; or have both unknown or missing race and unknown or missing ethnicity. §The jurisdictions used for the hospitalization calculation had a smaller percentage of AA or Black, NH; Asian or PI, NH; and Hispanic persons, and a larger percentage of White, NH; and AI or AN, NH, persons than the US 2019 population.

**Table 2:** Demographic and clinical characteristics of SARS-CoV-2 infections that met inclusion criteria for case fatality rate analysis, United States, 2020*

Characteristic	Includes cases for which death status was not known, counted as live				Includes only cases for which death status was known		
	No. deaths	No. cases	Case-fatality rate, %		No. deaths	No. cases	Case-fatality rate, %
Overall	78,663	4,708,444	1.7		78,663	3,952,311	2.0
Sex							
M	42,184	2,230,579	1.9		42,184	1,871,418	2.3
F	36,330	2,452,617	1.5		36,330	2,058,984	1.8
Other	0	45	0		0	42	0
Missing†	54	4,822	1.1		54	4,444	1.2
Unknown	95	20,381	0.5		95	17,423	0.6
Age group, y							
<1	10	21,331	0.05		10	18,694	0.05
1–4‡	7	66,098	0.01		7	58,590	0.01
5–14‡	15	270,467	0.01		15	233,021	0.01
15–24	175	864,837	0.02		175	717,778	0.02
25–34	571	849,838	0.06		571	717,833	0.08
35–44	1,290	717,446	0.2		1,290	605,432	0.2
45–54	3,390	687,837	0.5		3,390	576,674	0.6
55–64	8,701	589,496	1.5		8,701	488,624	1.8
65–74	16,197	341,936	4.7		16,197	283,996	5.7
75–84	21,689	180,869	12.0		21,689	150,871	14.4
>85	26,614	112,566	23.6		26,614	95,515	27.9
Missing†	4	5,723	0.06		4	5,283	0.08
Race and ethnicity§¶							
AI or AN, NH	591	30,312	2.0		591	24,687	2.4
Asian or PI, NH	2,847	96,073	3.0		2,847	86,842	3.3
AA or Black, NH	6,529	230,117	2.8		6,529	204,784	3.2
Hispanic	2,694	347,365	0.8		2,694	282,684	1.0
Other or multiple races, NH	2,270	148,408	1.5		2,270	133,038	1.7
White, NH	41,451	1,512,389	2.7		41,451	1,207,934	3.4
Unknown	22,281	2,343,780	1.0		22,281	2,012,342	1.1
Hospitalized#							
Yes	45,328	257,208	17.6		45,328	235,206	19.3
No	13,133	2,182,361	0.6		13,133	1,982,656	0.7
Unknown	9,809	1,116,549	0.9		9,809	652,037	1.5
Missing	10,393	1,152,326	0.9		10,393	1,082,412	1.0
Hospitalized or ICU**							
Hospitalized in ICU	13,906	31,461	44.2		13,906	30,122	46.2
Hospitalized, not ICU	31,497	226,235	13.9		31,497	205,428	15.3
Not hospitalized	13,058	2,181,873	0.6		13,058	1,982,312	0.7
Unknown	20,202	2,268,875	0.9		20,202	1,734,449	1.2
Symptom status							
Symptomatic	43,395	2,537,903	1.7		43,395	2,212,265	2.0
Asymptomatic	1,850	110,446	1.7		1,850	109,243	1.7
Missing†	12,504	814,456	1.5		12,504	672,665	1.9
Unknown	20,914	1,245,639	1.7		20,914	958,138	2.2

*Data from 22 jurisdictions that met the study inclusion criteria, Case Only Epi Task Force dataset, accessed March 17, 2021, based on responses to the CDC 2019 Novel Coronavirus Case Report Form during May 1–December 1, 2020 (Figure). Reports in which no response was provided about death were excluded from the rate calculation. AA, African American; AI, American Indian; AN, Alaska Native; NH, not Hispanic or Latino; ICU, intensive care unit; PI, Pacific Islander. †Missing indicates that the field was left blank. ‡Case-fatality rate was 0.0105%–0.0119% for 1–4 years age group and 0.0055%–0.0064% for 5–14 years age group. §Other or multiple races category includes NH persons whose race was reported as other or for whom >1 race was reported and persons whose record had both a racial designation and “racial information unknown” selected. The unknown category includes cases who have a known ethnicity of NH, but either unknown or missing race; have a known race but either unknown or missing ethnicity; or have both unknown or missing race and unknown or missing ethnicity. ¶The jurisdictions used for the fatality calculation had a greater percentage of Asian or PI, NH, persons and a smaller percentage of AA or Black, NH, persons than the US 2019 population. #Hospitalized status (yes or no) collected in a separate variable from “hospitalized or ICU” and numbers of hospitalized patients may not match. **Known to have been admitted to an ICU. The dataset contained separate variables for “hospitalized” and for “hospitalized in an ICU.” The 2 variables differed in the number not hospitalized and number hospitalized not in an ICU by <100 persons.

### Case-Hospitalization Rates

Twenty-one jurisdictions met the inclusion criteria for the case-hospitalization rate calculation. The overall case hospitalization rate among case-patients was 5.7%. The rate was <8.3% for every age group for persons up to 64 years of age. The rate was 5.0% for infants (defined as <1 year of age), 1.2% for children 1–4 years of age, and 0.6% for children 5–14 years of age. Among persons >15 years of age, the rate steadily increased for each older age group; it was 16.3% in persons 65–74 years of age and 25.9% in persons 75–84 and >85 years of age. Overall, the case hospitalization rate by sex was 6.2% for male and 5.2% for female. Female persons had lower case-hospitalization rates in every age group except those 15–24 and 25–34 years of age (Table 3). The case-hospitalization rate was 6.3% for persons whose report indicated they had symptoms and 3.3% for persons whose report indicated they were asymptomatic (Table 1).

**Table 3:** Case-hospitalization rates by age group and sex of patients with SARS-CoV-2 infections that met inclusion criteria for case hospitalization rate analysis, United States, 2020*

Age group, y	Case-hospitalization rate including cases for which hospitalization status was not known, counted as not hospitalized, %			Case-hospitalization rate among cases for which hospitalization status was known, %	
	Male sex	Female sex		Male sex	Female sex
<1	5.2	4.9		6.9	6.4
1–4	1.3	1.1		1.7	1.4
5–14	0.6	0.6		0.8	0.8
15–24	0.7	1.1		1.0	1.5
25–34	1.6	2.2		2.2	3.0
35–44	3.0	2.6		4.2	3.6
45–54	5.3	4.1		7.5	5.6
55–64	9.4	7.1		13.2	9.9
65–74	18.5	14.3		24.9	19.3
75–84	30.1	22.5		39.7	30.8
>85	35.4	21.4		47.8	31.7
Missing†	1.4	1.6		2.5	2.4

*Data from 21 jurisdictions that met the study inclusion criteria, CDC line level surveillance dataset, accessed March 17, 2021, based on responses to the CDC 2019 Novel Coronavirus Case Report Form during May 1–December 1, 2020. Reports in which no response was provided about hospitalization were excluded from the rate calculation. †Missing indicates that the field was left blank.

The crude case-hospitalization rate was highest among persons who were African American or Black, not Hispanic or Latino (11.4%). After age adjustment, the highest case-hospitalization rates were among persons who were African American or Black, not Hispanic or Latino (14.0%), and Asian or Pacific Islander, not Hispanic or Latino (11.2%); persons who were White, not Hispanic or Latino, had the lowest rate (6.8%) (Table 4).

**Table 4:** Race- and ethnicity-specific unadjusted and age-adjusted case hospitalization and fatality rates of patients with SARS-CoV-2 infections that met inclusion criteria for fatality rate analysis, United States, 2020*

Race and ethnicity†	Case hospitalization rate including cases for which hospitalization status was not known, counted as not hospitalized			Case fatality rate including cases for which death status was not known, counted as live	
	Unadjusted	Age-adjusted‡		Unadjusted	Age-adjusted‡
AI or AN, NH	7.4	10.1		2.0	3.4
Asian or PI, NH	7.0	11.2		3.0	4.4
AA or Black, NH	11.4	14.0		2.8	4.0
Hispanic	4.7	9.0		0.8	2.5
Other or multiple races, NH	7.8	9.8		1.5	2.1
White, NH	6.5	6.8		2.7	1.5
Unknown	3.4	4.2		1.0	2.6

*Data from 21 (hospitalization) and 22 (fatality) jurisdictions that met the study inclusion criteria, CDC line level surveillance dataset, accessed March 17, 2021, based on responses to the CDC 2019 Novel Coronavirus Case Report Form during May 1–December 1, 2020. Reports in which no response was provided about death or hospitalization were excluded from the rate calculation. AA, African American; AI, American Indian; AN, Alaska Native; ICU, intensive care unit; NH, not Hispanic or Latino; PI, Pacific Islander.. †Other or multiple races category includes not Hispanic or Latino persons whose race was reported as other or for whom >1 race was reported and persons whose record had both a racial designation and “racial information unknown” selected. The unknown category includes cases who have a known ethnicity of NH, but either unknown or missing race; have a known race but either unknown or missing ethnicity; or have both unknown or missing race and unknown or missing ethnicity. ‡Hospitalization and fatality rates by race and ethnicity adjusted to the age distribution of the White, NH, population based on 2019 US Census population.

### Case-Fatality Rates

Twenty-two jurisdictions met the inclusion criteria for the fatality rate calculation. The overall case-fatality rate was 1.7%. The rate was <1.6% for all age groups up to persons 64 years of age. The rate was 0.05% for infants and 0.01% for children 1–4 and 5–14 years of age. The 10 infants who died (6 girls) had laboratory-confirmed SARS-CoV-2 infection. Among persons >15 years of age, the rate steadily increased for each older age group; it was 4.7% in persons 65–74 years of age, 12.0% in persons 75–84 years of age, and 23.6% in persons >85 years of age (Table 2; Figure). Overall, the case-fatality rate was 1.9% for male sex and 1.5% for male sex (Table 2). Case-fatality rates for female sex were lower than or equal to those for male sex in every age group except infants (Table 5). The fatality rate was 1.7% both for persons whose report indicated they had symptoms and for persons whose report indicated they were asymptomatic (Table 2).

**Figure:**
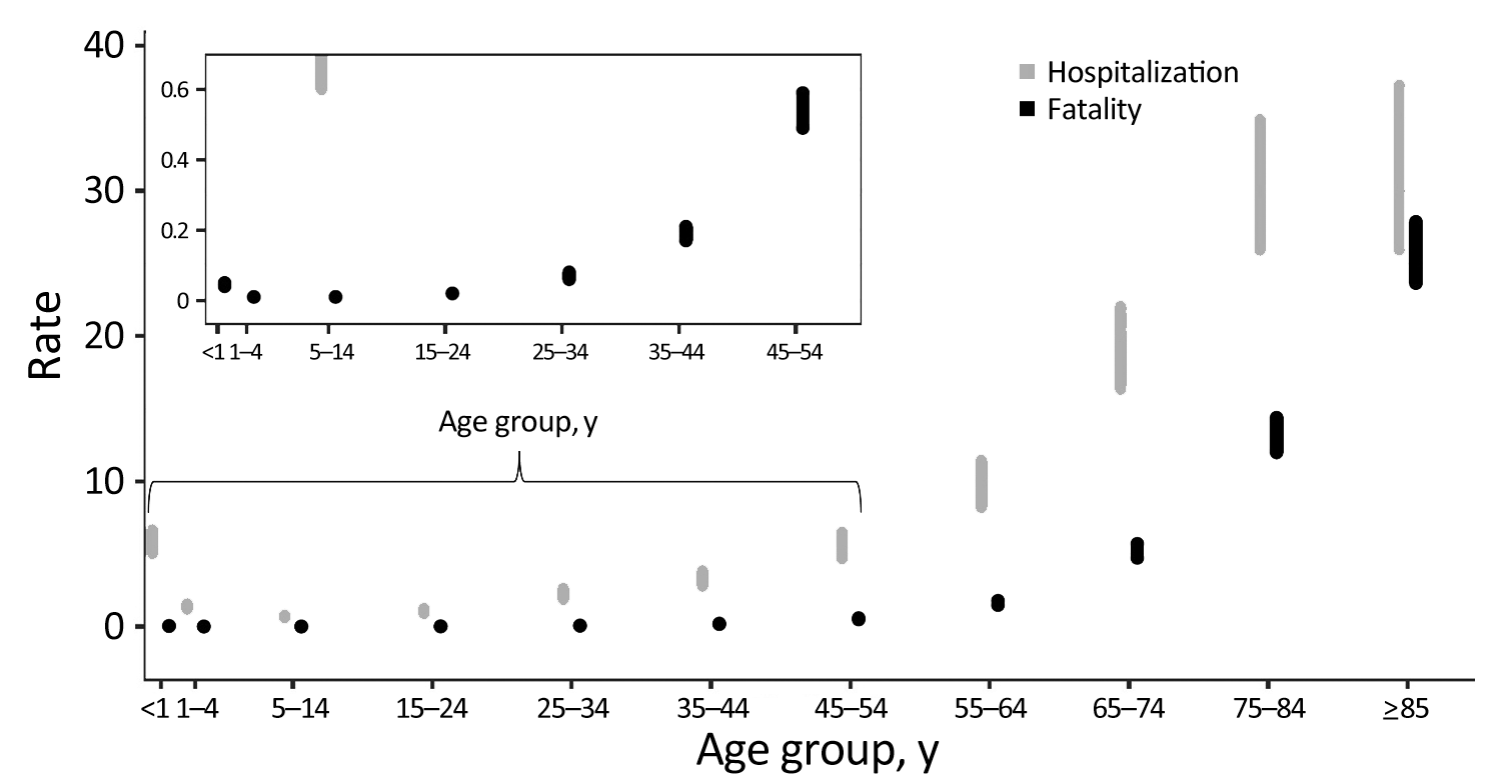
Upper and lower estimates of case-hospitalization (gray) and case-fatality (black) rates by age group of patients with SARS-CoV-2 infection, United States, 2020. Graphical representation of upper and lower estimates of rates in Tables 1 and 2. For case-hospitalization, lower bound was calculated by including cases with unknown hospitalization information as not hospitalized and upper bound by excluding cases with unknown hospitalization information. For case-fatality, lower bound was calculated by including cases with unknown death status as alive and upper bound by excluding cases with unknown death status information. Reports in which no response was provided about death or hospitalization were excluded from the respective rate calculation. Inset graph provides greater detail for younger age groups by using smaller y-axis values.

**Table 5:** Case-fatality rates by age group and sex of patients with SARS-CoV-2 infections, United States, 2020*

Age group, y	Case-fatality rates including cases for which death status was not known, counted as live, %			Case-fatality rates among cases for which death status was known, %	
	Male	Female		Male	Female
<1	0.04	0.06		0.04	0.07
1–4	0.01	0.01		0.01	0.01
5–14	0.00	0.01		0.01	0.01
15–24	0.03	0.01		0.03	0.02
25–34	0.09	0.05		0.11	0.06
35–44	0.2	0.1		0.3	0.2
45–54	0.7	0.3		0.8	0.4
55–64	2.0	1.0		2.4	1.2
65–74	6.0	3.6		7.2	4.3
75–84	14.7	9.8		17.5	11.8
>85	29.4	20.9		34.0	24.9
Missing†	0.1	0.0		0.1	0.0

*Data from 22 jurisdictions that met the study inclusion criteria, CDC line level surveillance dataset, accessed March 17, 2021, based on responses to the CDC 2019 Novel Coronavirus Case Report Form during May 1–December 1, 2020. Reports in which no response was provided about death were excluded from the rate calculation. †Missing indicates that the field was left blank.

The crude case-fatality rate was highest among persons who were Asian or Pacific Islander, not Hispanic or Latino (3.0%), followed by African American or Black, not Hispanic or Latino (2.8%), and White, not Hispanic or Latino (2.7%) (Table 2). After age adjustment, fatality rates among all racial and ethnic groups except White, not Hispanic or Latino, increased (Table 4): 4.4% for Asian or Pacific Islander, not Hispanic or Latino; 4.0% for African American or Black, not Hispanic or Latino; 3.4% for American Indian or Alaska Native, not Hispanic or Latino; 2.5% for Hispanic or Latino; and 2.1% for other or multiple races, not Hispanic or Latino. The rate decreased to 1.5% for persons who were White, not Hispanic or Latino. The fatality rate was 0.6% in persons who were not hospitalized, 17.6% among all persons who were hospitalized, and 44.2% in those admitted to an ICU (Table 2). Hospitalization and fatality rates by age group showed a steady increase, with highest rates among older age groups (Figure).

### Survey of Surveillance Practices

Health officials in 17 (65%) of the 26 jurisdictions included in either or both of the hospitalization and deaths final datasets responded to the survey. Officials in 6 jurisdictions commented that default values chosen for death reporting may be “no” or “alive” when the outcome is unknown. The methods used to determine if a death was COVID-19–related varied by jurisdiction; 7 reported using the CSTE COVID-19 case definition, and 10 reported using guidance unique to their state (e.g., designating death as COVID-19–related if a positive COVID-19 laboratory result was recorded in temporal proximity to the death).

## Discussion

We describe hospitalization and fatality rates of persons with COVID-19 reported through CDC surveillance, by age group, sex, race and ethnicity, and hospital and intensive care, during May 1–December 1, 2020, before the availability of COVID-19 vaccines and widespread commercial at-home testing. This study estimated rates in large US populations that included 2.4 million COVID-19 cases for measuring hospitalization rates and 4.7 million cases for fatality rates.

Age was a primary driver of SARS-CoV-2 hospitalization and death; rates had a U-shaped curve, being higher in infants, lowest in children 5 to 14 years of age, and highest among persons >65 years of age, confirming previous reports (*3*,*17*,*18*). Other studies have identified older age and underlying medical conditions as risk factors for severe COVID-19 outcomes, including hospitalization, admission to an ICU, requiring mechanical ventilation, and death (*3*,*18*). Those findings highlight the importance of vaccination and other mitigation methods to prevent SARS-CoV-2 infection in high-risk age groups.

Fatality rates were lowest for children 1–14 years of age (0.01%), in keeping with previous reports (*19*). We found fatality rates among infants to be slightly higher (0.05%). We have not found comparable data for infants in other studies. Higher fatality and hospitalization rates in infants may be attributable to other factors, including maternal COVID-19 infection or medical problems that may arise during the first year of life. Hospitalization rates and mortality rates are generally higher among infants than older children, primarily from complications of prematurity and birth defects (*20*,*21*). Our findings among infants should be interpreted with caution because only 10 deaths were reported among the 21,331 infant cases.

We found that male persons had higher hospitalization rates than female persons except in the 15–24- and 25–34-year age groups, and that male persons, except infants, had higher fatality rates than female persons. The reason for the disparity by sex is unknown; biologic, behavioral, and psychosocial factors could be involved (*22*–*26*). The 2 female age groups with higher hospitalization rates than the male group include female persons of childbearing age. Pregnancy and recent pregnancy may be associated with severe COVID-19 outcomes, including hospitalization and admission to ICUs (*27*,*28*).

Both unadjusted and age-adjusted hospitalization and fatality rates showed differences by race and ethnicity; the highest hospitalization rate was for the African American or Black, not Hispanic category, and the highest fatality rates were for the Asian or Pacific Islander and African American or Black, not Hispanic categories. Similar disparities in SARS-CoV-2 infections have been reported in other studies. Data in our analysis did not include information (e.g., socioeconomic status and occupation) to enable further examination of the reasons for these disparities (*7*). One study found no association between race and ethnicity category and severe COVID-19 outcomes after primary COVID-19 vaccination series (receipt of 2 doses), which suggests that access to COVID-19 vaccines can help mitigate racial and ethnic disparities (*18*).

We found that almost half of persons admitted to an ICU died, similar to studies of <400 persons conducted in Seattle (50%), Washington (52%), and New York City (78%) (*22*,*29*,*30*). This finding emphasizes the importance of early diagnosis and treatment of SARS-CoV-2 infection. Mechanical ventilation is often associated with high fatality rates (*22*,*26*,*31*).

One limitation of this analysis is the use of data from only 21 jurisdictions for hospitalization rate calculations and 22 for fatality rate calculations of the 56 jurisdictions that reported case information to CDC. However, the inclusion of only those jurisdictions is also a strength of the analysis because a relatively high percentage of case reports had valid, nonmissing hospitalization or death data. A high percentage of case reports were for persons with unknown race and ethnicity, in large part because reports without ethnicity were classified as unknown even if race was recorded (*32*); a smaller percentage were categorized as having other or multiple races. Had ethnicity been known, the hospitalization rate for racial and ethnic groups with added cases would be lower, and the effect on death rate would vary by group. Public health authorities have made recommendations to improve race and ethnicity reporting in surveillance data (*33*,*34*). Our comparisons of age-adjusted rates among racial and ethnic groups do not adjust for differences in prevalence of underlying diseases. Tests were performed only on persons who sought testing or medical care; therefore, cases in persons with no or mild symptoms were less likely to be identified and reported. Differences in the application of death definitions across jurisdictions highlight the need for standardized definitions during a pandemic; in December 2021, CSTE released a definition of COVID-19–associated deaths, developed with input from jurisdictional health departments (*35*). Limitations of using surveillance data to estimate fatality rates include preferential ascertainment of severe infections that may lead to overestimation of fatality rates, and the effect of specific interventions (e.g., hospitalization or hospitalization at a particular hospital) on survival (*36*). Although the National Center for Health Statistics has provisional data on COVID-19–related deaths, it does not have information on ill persons who did not die, so rates cannot be calculated from these data. We used surveillance data, which commonly, and in this study, have quality issues, including missing key variables such as race, ethnicity, and symptoms. Because of those limitations, we bounded our primary results by different assumptions on which outcome those with missing data had and we avoided statistical approaches, which, because of our large sample size, would have led to misleadingly narrow confidence bounds. Furthermore, the large sample size ensures that many differences would be statistically significant even if those differences may be of little practical significance.

Caution is needed before drawing conclusions from our study population. One might expect concerns with the quality of data collected during an emergency response above those in other surveillance data. Yet, such surveillance data can provide information that would not otherwise be available (*37*). Despite the limitations of our study dataset, the inclusion of data from most of the country, including many persons of all ages, races, and ethnicities, and our method of accounting for missing information in the analyses make these estimates valuable to the public health community.

This analysis presents case-hospitalization and case-fatality rates by age group, sex, and racial and ethnic groups before the introduction of vaccinations targeting SARS-CoV-2. The introduction of vaccinations and the presence of new strains of SARS-CoV-2 altered those rates. Moreover, the wide availability of at-home tests for detecting infection and the lack of national reporting for home testing results make it more likely that only severe infections will be reported. Our results document the severity of SARS-CoV-2 infections early in the pandemic, provide a baseline for future comparisons, and highlight the importance of preventing severe illness in high-risk populations (e.g., through vaccination, early identification of symptoms, testing, and isolation to prevent transmission). Devising appropriate control measures, with community input, for persons in historically underserved racial and ethnic groups and among adults >65 years of age is especially important (*18*).

Appendix Additional information about estimates of SARS-CoV-2 hospitalization and fatality rates in the prevaccination period, United States.
