# Supplementary material for: Estimates of SARS-CoV-2 Hospitalization and Fatality Rates in the Prevaccination Period, United States
Source: Emerg Infect Dis. 2024 Jun;30(6):1144–53. doi: 10.3201/eid3006.231285 (PMC11138987; doi:10.3201/eid3006.231285)
Supplement: Appendix — Additional information about estimates of SARS-CoV-2 hospitalization and fatality rates in the prevaccination period, United States. [file 23-1285-Techapp-s1.pdf]

Article DOI: https://doi.org/10.3201/eid3006.231285

Estimates of SARS-CoV-2 Hospitalization and Fatality Rates in the Prevaccination Period, United States

Appendix

Council of State and Territorial Epidemiologists Case Definitions

The Council of State and Territorial Epidemiologists have updated the case definition for COVID-19 several times in response to new information about the disease, including during the period covered in this manuscript: Interim 20-ID-01 was in effect from April 5 to August 5, 2020, and Interim 20-ID-02 was in effect from August 5, 2020 through the end of the study period.

Interim-20-ID-01

Clinical Criteria for Coronavirus Disease 2019 (COVID-19): At least two of the following symptoms: fever (measured or subjective), chills, rigors, myalgia, headache, sore throat, new olfactory and taste disorder(s) or at least one of the following symptoms: cough, shortness of breath, or difficulty breathing or severe respiratory illness with at least one of the following: clinical or radiographic evidence of pneumonia, or acute respiratory distress syndrome (ARDS) and no alternative more likely diagnosis. A confirmed case was defined as a case that meets confirmatory laboratory evidence. A probable case met the clinical criteria and epidemiologic linkage without confirmatory testing performed, or had presumptive laboratory evidence and either clinical or epidemiologic criteria, or met vital records criteria with no confirmatory laboratory testing performed. CSTE Position Statement Interim-20-ID-01, approved April 5, 2020. Retrieved from https://ndc.services.cdc.gov/case-definitions/coronavirus-disease-2019-2020/

Interim-20-ID-02

Clinical criteria for Coronavirus Disease 2019 (COVID-19): In the absence of a more likely diagnosis: At least two of the following symptoms: fever (measured or subjective), chills,

rigors, myalgia, headache, sore throat, nausea or vomiting, diarrhea, fatigue, congestion or runny nose; -or any one of the following symptoms: cough, shortness of breath, difficulty breathing, new olfactory disorder, new taste disorder;-or severe respiratory illness with at least one of the following: clinical or radiographic evidence of pneumonia, acute respiratory distress syndrome (ARDS). A confirmed case was defined as a case that meets confirmatory laboratory illness. A probable case met the clinical criteria and epidemiologic linkage without confirmatory testing performed, or had presumptive laboratory evidence, or met vital records criteria without confirmatory laboratory evidence. CSTE Position Statement Interim-20-ID-02, approved August 5, 2020. Retrieved from: https://ndc.services.cdc.gov/case-definitions/coronavirus-disease-2019-2020-08-05.

Applicable Federal Law and CDC Policy

Department of Health and Human Services – 45 C.F.R. part 46, 21 C.F.R. part 56; 42 U.S.C. Sect. 241(d); 5 U.S.C. Sect. 552a; 44 U.S.C. Sect. 3501 et seq. Available from: https://www.hhs.gov/ohrp/sites/default/files/ohrp/policy/ohrpregulations.pdf. Accessed February 4, 2021. CDC Clearance STARS Project ID: 0900f3eb81c82100 and Accession ID: NCEZID-QBHS-1/23/21–82100.

See e.g., 45 C.F.R. part 46, 21 C.F.R. part 56; 42 U.S.C. §241(d); 5 U.S.C. §552a; 44 U.S.C. §3501 et seq.

Appendix Table 1. Comparison of demographic characteristics of 2019 U.S. Census population used to calculate hospitalization and fatality rates with study population subsets

Characteristic	U.S., 2019		State subset used to calculate hospitalization rates including cases for which hospitalization status was not known*		State subset used to calculate fatality rates including cases for which death status was not known†	
Population	328,239,523	100.0%	83,257,734	100.0%	155,275,086	100.0%
Sex						
Male	161,657,324	49.2%	41,047,270	49.3%	76,462,054	49.2%
Female	166,582,199	50.8%	42,210,464	50.7%	78,813,032	50.8%
Age group, y						
0	3,783,052	1.2%	952,852	1.1%	1,781,198	1.1%
1–4	15,793,631	4.8%	3,982,853	4.8%	7,401,826	4.8%
5–14	40,994,163	12.5%	10,360,414	12.4%	19,172,914	12.3%
15–24	42,687,510	13.0%	10,807,827	13.0%	20,167,049	13.0%
25–34	45,940,321	14.0%	11,453,532	13.8%	21,860,881	14.1%
35–44	41,659,144	12.7%	10,401,995	12.5%	19,612,276	12.6%
45–54	40,874,902	12.5%	10,195,455	12.2%	19,379,663	12.5%
55–64	42,448,537	12.9%	10,997,770	13.2%	20,234,141	13.1%
65–74	31,483,433	9.6%	8,301,621	10.0%	14,844,167	9.6%
75–84	15,969,872	4.9%	4,118,297	4.9%	7,495,300	4.8%
85+	6,604,958	2.0%	1,685,181	2.0%	3,235,671	2.1%
Race and ethnicity‡,§						
White, NH	200,853,409	61.2%	60,811,268	73.0%	96,230,159	62.0%
AA or Black, NH	43,235,803	13.2%	10,649,508	12.8%	18,388,559	11.8%
AI or AN, NH	2,765,443	0.8%	1,015,877	1.2%	1,262,342	0.8%
Asian or PI, NH	20,819,191	6.3%	3,373,615	4.1%	11,884,296	7.7%
Hispanic	60,565,677	18.5%	7,407,466	8.9%	27,509,730	17.7%
Other or Multiple races, NH‖	NA	NA	NA	NA	NA	NA
Unknown‖	NA	NA	NA	NA	NA	NA

*Data from 21 jurisdictions that met the study inclusion criteria, CDC line-level surveillance dataset, accessed 03/17/2021, based on responses to the CDC 2019 Novel Coronavirus Case Report Form for May 1 – December 1, 2020. Reports in which no response was provided about hospitalization were excluded.

†Data from 22 jurisdictions that met the study inclusion criteria, CDC line-level surveillance dataset, accessed 03/17/2021, based on responses to the CDC 2019 Novel Coronavirus Case Report Form for May 1 – December 1, 2020. Reports in which no response was provided about death were excluded.

‡AI or AN, NH = American Indian or Alaska Native, not Hispanic or Latino; Asian or PI, NH = Pacific Islander, not Hispanic or Latino; AA or Black, NH = African American or Black, not Hispanic or Latino; Hispanic = Hispanic; Other or Multiple races, NH = Multiple or other, not Hispanic or Latino; White, NH = White, not Hispanic or Latino. Other or Multiple races category includes not Hispanic or Latino persons whose race was reported as other or for whom more than one race was reported and persons whose record had both a racial designation and “racial information unknown” selected. The Unknown category includes cases who have a known ethnicity of not Hispanic or Latino, but either unknown or missing race; have a known race but either unknown or missing ethnicity; or have both unknown or missing race and unknown or missing ethnicity.

§The jurisdictions used for the hospitalization calculation had a smaller percentage of Black, not Hispanic or Latino; Asian or Pacific Islander, not Hispanic or Latino; and Hispanic persons, and a larger percentage of White, not Hispanic or Latino and American Indian or Alaska Native, not Hispanic or Latino persons than the U.S. 2019 population.

‖ NA = Not available; 2019 U.S. Census does not have Race/Ethnicity data on “Unknown” or “Other or Multiple Races, NH”

Appendix Table 2. Demographic description of all reported COVID-19 cases, May 1 - December 1, 2020, United States

Characteristic	No. cases	%
Overall	10,332,323	100.0%
Sex		
Male	4,866,732	47.1%
Female	5,356,123	51.8%
Other	209	0.0%
Missing [†]	22,123	0.2%
Unknown	87,136	0.8%
Age group, y		
0	47,101	0.5%
1–4	144,632	1.4%
5–14	578,870	5.6%
15–24	1,864,002	18.0%
25–34	1,860,091	18.0%
35–44	1,584,888	15.3%
45–54	1,515,156	14.7%
55–64	1,289,692	12.5%
65–74	761,792	7.4%
75–84	401,749	3.9%
85+	238,978	2.3%
Missing [†]	45,372	0.4%
Race and ethnicity[‡]		
AI or AN, NH	76,579	0.7%
Asian or PI, NH	183,924	1.8%
AA or Black, NH	701,304	6.8%
Hispanic	1,331,989	12.9%
Other or Multiple races, NH	281,893	2.7%
White, NH	3,202,049	31.0%
Unknown	4,554,585	44.1%
Hospitalized [Π]		
Yes	484,436	4.7%
No	4,221,863	40.9%
Unknown	1,373,215	13.3%
Missing[†]	4,252,809	41.2%
Hospitalized/Intensive Care Unit[#]		
Hospitalized in ICU	44,847	0.4%
Hospitalized, not ICU	440,365	4.3%
Not hospitalized	4,221,087	40.9%
Unknown/Missing	5,626,024	54.5%
Symptom status		
Symptomatic	4,763,116	46.1%
Asymptomatic	188,924	1.8%
Missing[†]	4,030,090	39.0%
Unknown	1,350,193	13.1%
Death		
Yes	175,669	1.7%
No	5,360,160	51.9%
Unknown	1,025,955	10.0%
Missing[†]	3,770,539	36.5%

[†]Missing indicates that the field was left blank.
[‡]AI or AN, NH = American Indian or Alaska Native, not Hispanic or Latino; Asian or PI, NH = Pacific Islander, not Hispanic or Latino; AA or Black, NH = African American or Black, not Hispanic or Latino; Hispanic = Hispanic; Other or Multiple races, NH = Multiple or other, not Hispanic or Latino; White, NH = White, not Hispanic or Latino. Other or Multiple races category includes not Hispanic or Latino persons whose race was reported as other or for whom more than one race was reported and persons whose record had both a racial designation and "racial information unknown" selected. The Unknown category includes cases who have a known ethnicity of not Hispanic or Latino, but either unknown or missing race; have a known race but either unknown or missing ethnicity; or have both unknown or missing race and unknown or missing ethnicity.
[Π] Hospitalized status (yes/no) collected in a separate variable from Hospitalized/ICU and numbers of hospitalized patients may not match.
[#] Known to have been admitted to an intensive care unit. The dataset contained separate variables for "hospitalized" and for "hospitalized in an ICU." The two variables differed in the number not hospitalized and number hospitalized not in an ICU differ by fewer than 800 persons.
